# The Thirty-Fourth Annual Commencement of the Baltimore College of Dental Surgery

**Published:** 1874-03

**Authors:** 


### EDITORIAL, ETC.

---

*The Thirty-fourth Annual Commencement of the Baltimore College of Dental Surgery* was held at Concordia Opera House, on Thursday evening, February 26th, 1874, in the presence of so large an audience that the spacious hall was crowded to such a degree that many were obliged to stand during the entire exercises. The right of the stage was occupied by the Faculty of the College, a number of the members of the Faculties of the different Medical Colleges of the city, and many invited guests, among the latter, alumni of the college from several Southern States. The left of the stage was occupied by the members of the graduating class, while the front along the foot-lights, was covered by a profusion of handsome boquets from the lady friends of the graduates, the number of which kept the members of the boquet committee in active employment for some time. The music was furnished by Prof. Minnick's orchestra.

The exercises commenced with prayer by the Rev. E. R. Eschbach, after which Professor F. J. S. Gorgas, Dean of the Faculty, in announcing the names of the graduates, and the authority by which the degrees were conferred, stated that since the organization of the Baltimore College of Dental Surgery, thirty-five years ago, nearly *eight hundred* graduates have received the degree of Doctor of Dental Surgery, and *twelve hundred* students have attended the lectures of the institution ; this College being the first, and for many years the only Dental College in the world.

The Degree of "Doctor of Dental Surgery" was then conferred by Professor Gorgas upon the following graduates, representing eight different States and Europe :

| | |
|---|---|
| John Abner Chapple, - - - - | Georgia. |
| Lewis Mileston Cowardin, - - - | Virginia. |
| Thomas H. Davy, - - - - | Maryland. |

| | |
|---|---|
| Henry Clay Devilbiss, - - - | Maryland. |
| Alfred Eubank, - - - - - | Alabama. |
| John W. Farmer, M. D., - - - | Virginia. |
| Homer Kenyon Green, - - - | Pennsylvania. |
| Louise Jacobi, - - - - - | Germany. |
| George Vernon Jenkins, - - - | Maryland. |
| Douglas Malcolm, - - - - | Maryland. |
| Charles Augustus Mercer, - - - | Virginia. |
| J. Henry Morgan, - - - - | Virginia. |
| James Bruce Moseley, - - | South Carolina. |
| David N. Rust. - - - - - | Virginia. |
| Thomas L. Sydnor, - - - - - | Virginia. |
| Thomas Ritchie Vermillion, - - | Virginia. |
| Charles Ferdinand Wagner, M. D., - - | Germany. |
| William B. Wise, - - - - | Virginia. |
| Silas Robert Wyse, - - - - | Mississipi. |

After the degrees were conferred, during which ceremony the services of the Janitor of the College were required to assist in conveying the boquets from the stage, (some of the graduates receiving so many that baskets were required to contain them,) the Valedictory Address was delivered by Professor Henry R. Noel, M. D., of the Faculty. The subject of this Address was "The Mystery of Life," showing its relation and dependence upon the amimal kingdom, and was an interesting and scientific production. As we expect to publish this address in the Journal, our readers will have an opportunity for reading it.

The Class Address was delivered by John W. Farmer, M. D. of Virginia, and was appreciated by his class-mates and the Faculty of the College for its excellence.

The following were the officers of the graduating class of 1874. President, H. Clay Devilbiss, of Md. Vice President, D. Malcolm, of Md. Secretary, J. A. Chapple, of Ga. Treasurer, G. V. Jenkins, of Md. Committee of Arrangements.—G. V. Jenkins, W. B. Wise, C. A. Mercer, S. R, Wyse, T. H. Davy, H. R. Green and Thos. L. Sydnor. The exercises closed with the benediction by the Rev. Dr. Eschbach, and thus ended another of the sessions of the *alma mater* of so many of the dental practitioners of this and foreign countries.

The following changes have occurred in the Faculty of the

Baltimore College of Dental Surgery since the last annual commencement. Prof. M. J. DeRosset having resigned the chair of Chemistry, on account of his removal to Wilmington, N. C., Prof. W. P. Tonry was appointed Lecturer on Chemistry for the session just ended, a position which he filled to the satisfaction of both Faculty and Students. Prof. P. H. Austen having recovered from his recent illness, will fill the chair of Dental Science and Chemistry. Dr. James B. Hodgkin, of Alexandria, Va., was elected Professor of Mechanical Dentistry in October last.

During the coming spring and summer the College Building will be greatly enlarged by the addition of an adjoining building under a new common front, which will add to the size of both Lecture rooms, Infirmary and Dissecting room, and present an appearance not inferior to any institution of the kind in this country.

The *Thirty-fifth* Annual Session will commence on the 15th of October, 1874, and continue until March, 1875.

The Infirmary of the College is open during the entire year.

